# A Clinically Relevant Dosage of Mitoxantrone Disrupts the Glutathione and Lipid Metabolic Pathways of the CD-1 Mice Brain: A Metabolomics Study

**DOI:** 10.3390/ijms241713126

**Published:** 2023-08-23

**Authors:** Ana Dias-Carvalho, Ana Margarida-Araújo, Ana Reis-Mendes, Catarina Oliveira Sequeira, Sofia Azeredo Pereira, Paula Guedes de Pinho, Félix Carvalho, Susana Isabel Sá, Eduarda Fernandes, Vera Marisa Costa

**Affiliations:** 1 Associate Laboratory i4HB, Institute for Health and Bioeconomy, Faculty of Pharmacy, University of Porto, 4050-313 Porto, Portugal; 2 UCIBIO—Applied Molecular Biosciences Unit, Laboratory of Toxicology, Department of Biological Sciences, Faculty of Pharmacy, University of Porto, 4050-313 Porto, Portugal; 3 iNOVA4Health, LS4Future, NOVA Medical School|Faculdade de Ciências Médicas (NMS|FCM), Universidade Nova de Lisboa, 1150-082 Lisboa, Portugal; 4 Unit of Anatomy, Department of Biomedicine, Faculty of Medicine, University of Porto, 4200-319 Porto, Portugal; 5 Center for Health Technology and Services Research (CINTESIS), Faculty of Medicine, University of Porto, 4200-319 Porto, Portugal; 6 LAQV-REQUIMTE, Laboratory of Applied Chemistry, Department of Chemical Sciences, Faculty of Pharmacy, University of Porto, 4050-313 Porto, Portugal

**Keywords:** chemotherapy, adverse outcome pathways, cancer, multiple sclerosis, topoisomerase inhibitors, metabolomics approach

## Abstract

Long-term cognitive dysfunction, or “chemobrain”, has been observed in cancer patients treated with chemotherapy. Mitoxantrone (MTX) is a topoisomerase II inhibitor that binds and intercalates with DNA, being used in the treatment of several cancers and multiple sclerosis. Although MTX can induce chemobrain, its neurotoxic mechanisms are poorly studied. This work aimed to identify the adverse outcome pathways (AOPs) activated in the brain upon the use of a clinically relevant cumulative dose of MTX. Three-month-old male CD-1 mice were given a biweekly intraperitoneal administration of MTX over the course of three weeks until reaching a total cumulative dose of 6 mg/kg. Controls were given sterile saline in the same schedule. Two weeks after the last administration, the mice were euthanized and their brains removed. The left brain hemisphere was used for targeted profiling of the metabolism of glutathione and the right hemisphere for an untargeted metabolomics approach. The obtained results revealed that MTX treatment reduced the availability of cysteine (Cys), cysteinylglycine (CysGly), and reduced glutathione (GSH) suggesting that MTX disrupts glutathione metabolism. The untargeted approach revealed metabolic circuits of phosphatidylethanolamine, catecholamines, unsaturated fatty acids biosynthesis, and glycerolipids as relevant players in AOPs of MTX in our in vivo model. As far as we know, our study was the first to perform such a broad profiling study on pathways that could put patients given MTX at risk of cognitive deficits.

## 1. Introduction

Chemotherapy is still one of the most used anticancer treatments, as it has high efficacy rates. Nevertheless, it presents a long list of short- and long-term adverse effects that significantly impact the post-treatment quality of life. Chemotherapy-treated patients may experience significant cognitive impairment, which is commonly called “chemobrain” or “chemofog”, describing the cognitive dysfunction that results from systemic chemotherapy [1]. Chemobrain can affect up to 75% of treated patients [2]. The symptoms of cognitive impairment in chemotherapy-treated patients often come as deficits in attention, concentration, working memory, and executive function. Although the brain microenvironment is tightly protected by the blood–brain barrier (BBB), which is thought to prevent the passage of most anticancer drugs, clinical data suggest that chemotherapy can harm the brain [3].

Mitoxantrone (MTX) is a synthetic anthracenedione closely related to the anthracycline antitumoral class. MTX has antineoplastic and immunomodulatory effects [4,5], being used in the treatment of various cancers, including prostate, ovarian, stomach, and liver [5], and it was approved by the Food and Drug Administration (FDA) for the treatment of worsening relapsing-remitting multiple sclerosis, secondary progressive multiple sclerosis, and progressive-remitting multiple sclerosis [6]. MTX has cytostatic proprieties because of its ability to intercalate into DNA, forming stable adducts, interfering with RNA by blocking the RNA polymerase progression and inhibiting topoisomerase II [7]. Regarding its immunomodulatory effects, MTX inhibits T and B lymphocytes and macrophages, and it can inhibit the synthesis of pro-inflammatory cytokines [8,9]. Anticancer treatment with MTX consists of multiple intravenous administrations in a recommended dosage schedule of 12–14 mg/m^2^ every 3 to 4 weeks [10]. In multiple sclerosis treatment, the recommended intravenous dosage schedule is 12 mg/m^2^ every 3 months. Nevertheless, the total lifetime cumulative dose of 140 mg/m^2^ [5] should not be surpassed in anticancer treatment and 100 mg/m^2^ in multiple sclerosis patients [6].

Studies concerning MTX neurotoxicity are limited. However, in a retrospective longitudinal study of a cohort of patients with advanced prostate cancer that received chemotherapeutic agents, including MTX, it was shown that those patients were more likely to have central nervous System (CNS) events (amnesia or memory impairment, anxiety, ataxia, cognitive disorders, confusion, convulsions, disturbance in attention, dizziness, falls, fatigue/asthenia, hallucinations, headaches, insomnia, pain, paraesthesia, seizures, and weakness) than patients that received targeted therapy [11]. In another study [12], the cognitive function was evaluated in 20 patients with acute myeloid leukaemia, who first received cycles of daunorubicin and cytarabine. In the cases where remission was not achieved, the patients received cycles of MTX, etoposide, and cytarabine. The evaluation was performed before the start of the MTX treatment and at 1, 4, 6, 9, and 12 months post-treatment using the EORTC-QLQ30 questionnaire. The study concluded that patients’ scores on the cognitive subscale of the EORTC-QLQ30 were comparable to the published norms at all assessment time points [12]. Nevertheless, these studies do not evaluate solely the influence of MTX in cognition and use very broad questionnaires to access cognitive function. In another study, when MTX was given locally within a combination treatment against glioblastoma, short-lived partial Jacksonian motor seizures, and headache occurred, among other slight neurological symptoms [13].

Furthermore, MTX entrance through the BBB was shown to be limited by the action of ATP-binding cassette efflux transporters (namely, the breast cancer resistance protein (BCRP)) [14]. Therefore, is almost unlikely that MTX could reach the brain in clinically relevant doses in an intact BBB [15], but a disrupted BBB is expected in multiple sclerosis [16]. Furthermore, it is possible that non-BBB-crossing chemotherapeutic agents can induce neurotoxicity after acting on peripheral factors, namely, by causing oxidative stress and inflammation [1]. MTX is not as prone to generate reactive oxygen species (ROS), unlike many other chemotherapeutic agents. Nevertheless, the anthracenedione structure generates superoxide radicals and hydrogen peroxide that, in turn, might react with other cellular components [17] and may also oxidize blood serum proteins [18]. Furthermore, despite its immunosuppressive properties, MTX might promote inflammation in nontarget organs [19].

In a previous study, we demonstrated that a clinically relevant cumulative dose of MTX induces oxidative stress, apoptosis, and autophagy in the brain of adult male CD-1 mice [20]. Thus, in the present study, we aimed to further gain in-depth knowledge on the adverse outcome pathways (AOPs) of MTX, mainly the effect on redox homeostasis and general metabolic circuitries in brain resourcing to untargeted and targeted metabolomic approaches.

## 2. Results

### 2.1. MTX Affected the Glutathiolomic Profile, Globally Decreasing They Availability

**Figure 1:**
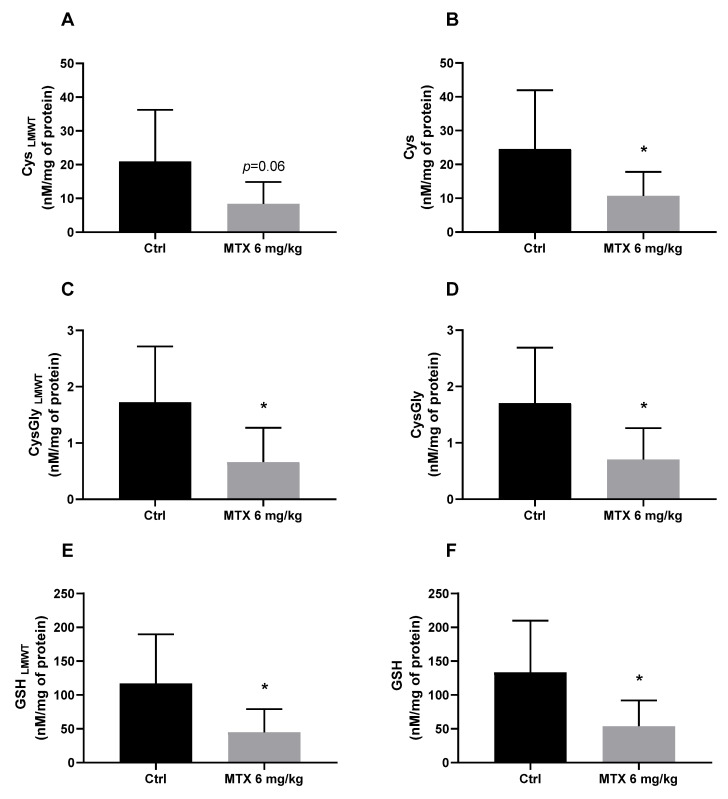
Evaluation of the gluthiolomic profile of MTX-treated mice: (**A**) Cys (Low molecular weight thiols) _LMWT_; (**B**) Cys; (**C**) Cys-Gly _LMWT_; (**D**) Cys-Gly; (**E**) GSH _LMWT_; (**F**) GSH levels, expressed in nM/mg of protein. The results are the mean ± SD from 8 animals in the control group (Ctrl) group and 9 animals in the MTX 6 mg/kg group. Statistical comparisons were made using the t-test analysis with Welch’s correction (* *p* < 0.05 vs. control).

A cumulative dose of 6 mg/kg of MTX caused significant alterations in the brain cysteine-related thiolomic profile. All analysed analytes significantly decreased (or tended to decrease) after MTX treatment (Figure 1A–F).

### 2.2. Global Endogenous Metabolic Profiling Shows That MTX Impacts Brain Metabolome

**Figure 2:**
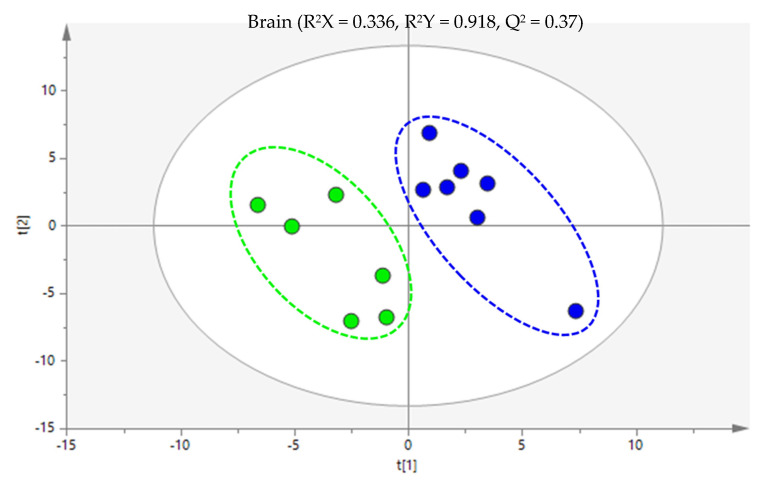
Partial least squares discriminant analysis (PLS-DA) score scatter plot of the 6 mg/kg MTX-treated mice (dark blue) in comparison to the controls (green). Each sample is represented in the score scatter plot as an individual variable. The ellipses indicate the 95% confidence limit of the models.

Partial least squares discriminant analysis (PLS-DA) was performed between the MTX-treated and the control animals (Figure 2). The model explains 91.8% of the variability between the groups and presented robust discrimination with predictive power (Q^2^ = 0.370). The cross-validation parameters obtained for the permutated PLS-DA model validated the initial model, since the values obtained in the permutation test were lower (R^2^ = 0.909 and Q^2^ = 0.164) than the values of the original model. After the multivariate analysis, a total of 27 metabolites were pointed out as potentially discriminant (|*p*(corr)| ≥ 0.5 and VIP > 1) between the brains of the control and MTX-treated mice. The Supplementary Materials Table S1 contain a comprehensive list detailing the general characteristics of all potential discriminant metabolites.

### 2.3. MTX Treatment Altered Several Brain Metabolic Pathways, Namely, the Biosynthesis of Phosphatidylethanolamine, Acid Ascorbic, Unsaturated Fatty Acids, and Glycerolipids Metabolism

The univariate analysis revelated that, of the 27 potentially discriminant metabolites, 5 metabolites (phosphorylethanolamine, ascorbic acid, palmitic acid, linoleic acid, and cholest-7-en-3-ol, (3β,5α)) were significantly altered in the MTX-treated mice in comparison to the control group, and 3 metabolites presented a tendency (*p* < 0.07; L-5-oxoproline, oleic acid, and 2-oleoylglycerol). The list of aforementioned metabolites and statistical characteristics, as well as potential biochemical pathways (whenever possible), are shown in Table 1. The upregulated metabolites (increased in comparison to the control group) include L-5-oxoproline, phosphorylethanolamine, ascorbic acid, and 2-oleoylglycerol and the downregulated metabolites (decreased in comparison to the control group) were palmitic acid, linoleic acid, oleic acid, and cholest-7-en-3-ol, (3β,5α).

**Figure 3:**
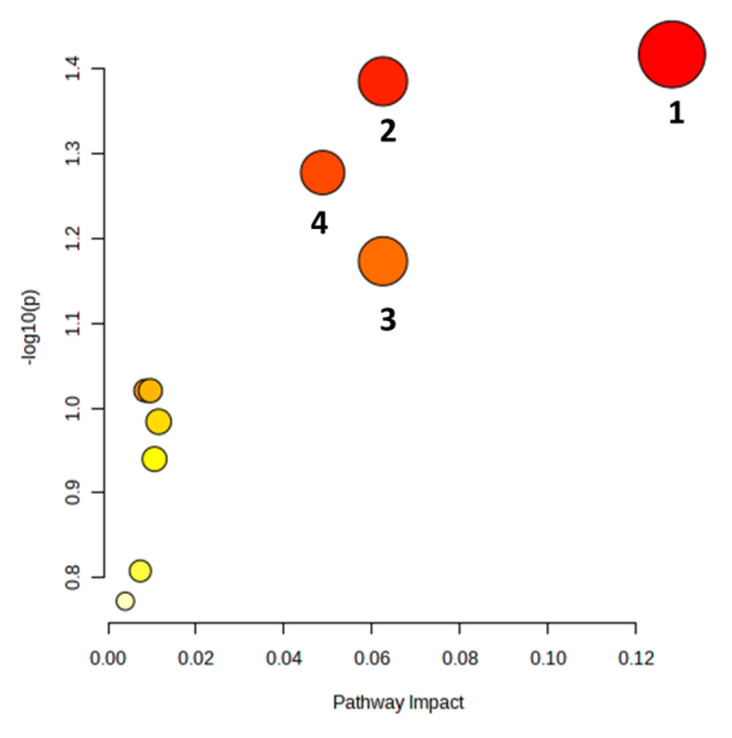
Summary of the brain’s metabolic pathways altered by a total cumulative dose of 6 mg/kg of MTX. The colour of each node is determined by its *p*-value, with darker shades representing a more significant pathway. The radius of the node reflects the pathway’s impact value. Altered pathways (*p* < 0.07) are numbered as follows: 1—phosphatidylethanolamine biosynthesis, 2—catecholamine biosynthesis, 3—glycerolipids metabolism, 4—biosynthesis of unsaturated fatty acids.

Using the MetaboAnalyst 5.0 software was possible to observe that the most dysregulated biochemical pathways induced by MTX were phosphatidylethanolamine biosynthesis, catecholamine biosynthesis, glycerolipids metabolism, and biosynthesis of unsaturated fatty acids (Figure 3).

## 3. Discussion

MTX neurotoxicity has been poorly investigated despite its long use in cancer treatment and recently multiple sclerosis. Nevertheless, chemotherapy-induced cognitive dysfunction significantly affects the post-treatment quality of patients’ life [21]. To the best of our knowledge, this is the first study to access the brain’s glutathione metabolism and to identify the brain profile of metabolites associated with treatment with a clinically relevant dose of MXT in CD-1 mice. The major findings of this work were that MTX-treatment decreased the brain’s thiols availability and significantly altered the brain’s endogenous metabolic profile, including disruption of the biosynthesis of phosphatidylethanolamine, catecholamines, and unsaturated fatty acids in addition to glycerolipids metabolism.

The brain is particularly susceptible to oxidative stress because of its high metabolic activity and high oxygen consumption. On the other hand, it has relatively low levels of antioxidant enzymes and compounds that could scavenge and neutralize ROS and prevent oxidative damage [22]. Oxidative stress caused by chemotherapeutic agents has been acknowledged as a potential cause of chemobrain, and although MTX does not induce high levels of oxidative stress in targeted organs, it can disrupt antioxidant defences [17,23]. Maintaining a robust antioxidant defence is essential to cope with oxidative stress and protect the brain from neurotoxicity. The glutatiolomic profile was obtained by the quantification of glutathione (GSH), its precursor cysteine (Cys), and its catabolism product cysteinylglicine (CysGly). For each thiol, both the non-protein-bound (free, low-molecular weight thiol LMWT) form and protein bound form (*S*-thiolated proteins, RSSP) were obtained. Therefore, the effect of MTX in 6 thiol pools were investigated, namely, GSH_LMWT_, Cys_LMWT_, CysGly_LMWT_, and S-glutathionylated (GSSP); S-cysteinylated (CysSSP); and S-cysteinylglicinylated (CysGlySSP) proteins, as in a earlier work of the group [24]. The tripeptide GSH (γ-glutamyl-cysteinyl-glycine) is one of the most abundant intracellular antioxidants and it is the thiol compound with the highest concentration in all cells of all organs [22]. GSH is synthesized in a two-step reaction: the first step is the condensation of Cys and glutamate to form γ-glutamylcysteine, and the second step involves the addition of glycine to γ-glutamylcysteine to form GSH [25]. GSH and related LMWT can bind to proteins through a disulphide bound [26]. The catabolism of GSH involves a series of enzymatic reactions, with the main pathways as follows: γ-glutamyl transpeptidase that forms y-glutamyl amino acids; dipeptidase that cleaves GSH to form CysGly; and direct oxidation to form glutathione disulphide (GSSG) [27]. This cycle of synthesis and degradation of glutathione is known as the γ-glutamyl cycle and plays a critical role in the regulation of cellular glutathione levels and the detoxification of ROS [28]. Furthermore, interaction between neurons and astrocytes is essential for GSH, co-culture of neurons and astrocytes demonstrated that GSH neuronal levels increase in the presence of astrocytes [29]. The analysis of the glutatiolomic profile performed in this study revealed that a total cumulative dose of 6 mg/kg of MTX overall affected the brain metabolism of glutathione. In this study, the precursor Cys, which is the limiting step in the synthesis of GSH [30], presented a tendency to decrease (*p* = 0.06) in the free form after MTX treatment and a significant decrease in the total assessment. Cys may also be catalysed by cystathionine-β-synthase, an enzyme expressed in astrocytes, and produce endogenous hydrogen sulphide (H_2_S) which, in turn, acts as a neuroprotective agent by protecting the neurons from apoptosis [31]. Therefore, MTX-induced depletion of Cys, not only compromises the synthesis of GSH but, also, affects the synthesis of neuroprotective agents. In this study, it was also seen that the total cumulative dose of 6 mg/kg also decreases CysGly both in the free and total form. Given the cyclic characteristics of GSH production, CysGly is not only a catabolic product of GSH but also an important exogenous precursor for the neuronal synthesis of GSH, since it is broken down by an aminopeptidase into Cys and glycine, and the obtained Cys enter the neurons to continue the cycle [32]. The measurement of the brain levels of GSH revealed that the MTX treatment caused a decrease in both the free and total forms. In a previous study, our group demonstrated that MTX (also with a total cumulative dose of 6 mg/kg) caused a tendency for a decrease of brain GSH and total glutathione levels one week after the last administration [20]. The methodology used in the presented study, and an increased number of animals or elapsed time, allowed us to observe more significant changes. GSH depletion indicates that MTX disrupted the antioxidant defences in the brain of treated mice, which could contribute to the neuronal damage observed in MTX-treated mice.

Metabolomics approaches represents a high-throughput analysis to identify changes in the quantity and profile of low-molecular metabolites (<1500 Da) in a biological system [33]. Given the lack of research into the neurotoxicity of MTX, the present work used an untargeted metabolic approach to simultaneously study various metabolites that might be altered after MTX treatment and provide valuable insights into related AOPs.

In this study, MTX treatment induced an increase in the brain levels of L-5-oxoproline. This metabolite, also known as pyroglutamic acid, is an intermediate in the γ-glutamyl cycle, the aforementioned pathway for the synthesis and degradation of GSH [34]. As the total cumulative dose of 6 mg/kg of MTX significantly depleted the levels of Cys, CysGly, and GSH, all participants in the γ-glutamyl cycle, L-5-oxoproline is going to accumulate since the cycle is compromised. Accumulation of L-5-oxoproline causes oxoprolinemia (or pyroglutamic acidaemia), and patients with this condition display significant cognitive dysfunction and atrophy of the cerebellum and lesions in the cortex and thalamus [35,36]. Therefore, MTX treatment depletes GSH and causes neurotoxic accumulation of L-5-oxoproline, which requires further studies as a relevant biomarker of cognitive dysfunction.

After a cumulative dose of 6 mg/kg, there was a significant disruption of the pathway of phosphatidylethanolamine biosynthesis, as seen by an increase in the levels of phosphorylethanolamine, a metabolite involved in, as well as a major component of, membrane biogenesis. Nevertheless, also considering the role of phosphorylethanolamine in autophagy, one cannot ignore its involvement in other pathways. This metabolite is essential for the synthesis of glycosylphosphatidylinositol-anchored proteins (GPI-APs) which, in turn, can form a covalent attachment to autophagy-related protein 8 (ATG8) and lead to autophagosome formation [37]. Our group has previously demonstrated that a cumulative dose of 6 mg/kg of MTX induced neuronal autophagy, as seen by the increase in microtubule-associated protein 1 light chain 3 (LC3), which is part of the ATG8 subfamily, and in p62 [20]. The upregulation observed in metabolomic analysis further confirms that MTX can induce neuronal autophagy. Prolonged autophagy may lead to the loss of essential cellular components, potentially compromising neuronal integrity and function, leading to neuronal loss, impacting the neuronal network essential for cognitive functioning [1,20].

Another striking observation in this study was the increase in the levels of ascorbic acid. Primates lack ascorbate biosynthesis capacity, as they have a deficiency in L-gulono-1,4-lactone oxidase expression, but mice are able to synthesize it, and making comparisons between rodent models and humans is not so straightforward on this subject [38]. Ascorbic acid (also known as vitamin C) is an antioxidant molecule and a cofactor for the enzyme dopamine beta-hydroxylase, which converts dopamine to noradrenaline [39]. However, studies have shown that ascorbic acid has a much greater role in neurotransmitter modulation, including controlling the levels of several bioenergetic amines [38] and inducing redox changes to activate the N-methyl-D-aspartate (NMDA) receptor protecting neurons from glutamate-induced excitotoxicity [40,41]. Therefore, the observed increase in ascorbic acid could be a compensatory mechanism to cope with excitotoxicity [42]. There is no literature available on the effects of MTX on brain catecholamine levels; however, impaired catecholamine synthesis could be responsible for the cognitive deficits observed in chemotherapy-treated patients. Nevertheless, the role of ascorbic acid and possible supplementation in humans requires further research to evaluate efficiency and efficacy.

The brain of MTX-treated mice also presented decreased levels of palmitic acid, indicating abnormal glycerolipids metabolism. Palmitic acid is a saturated fatty acid essential for brain function, since it is used by the neuronal cells as an energy source and is a precursor of signalling molecules [43]. Furthermore, palmitic acid is the substrate for the synthesis of more complex fatty acids that are needed for the process of myelination and remyelination by oligodendrocytes [44]; if this substrate is not available, it could compromise neuronal functioning. A study exploring the lipidome composition of the prefrontal cortex of 396 healthy individuals (ages spanning 100 years) and also 67 individuals with autism, schizophrenia, or Down’s syndrome, determined that individuals of all three diseases had decreased glycerophospholipid metabolism, which might be a common factor in cognitive dysfunction [45]. Furthermore, glycerophospholipid metabolism has been implicated in neuronal dendrite branching and outgrowth [46], and the downregulation of this pathway could compromise neuronal networks. MTX treatment also caused decreased brain levels of linoleic acid, suggesting dysfunction of the biosynthesis of unsaturated fatty acids. Linoleic acid is an essential n-6 polyunsaturated fatty acid. In adult (2 months) rats administrated with labelled linoleic acid (intravenously), it was observed that the majority (at least 86%) of the labelled linoleic acid that entered the brain is rapidly subjected to β-oxidation [47]. Only a small portion remains unchanged and is incorporated into various lipids, including acetyl coenzyme A, triglycerides, and phospholipids [47]. A decrease in the brain levels of linoleic acid after MTX treatment, as we see herein, might suggest a higher turnover of linoleic acid via β-oxidation. Nevertheless, this is not a preferred source of energy for the brain, since it demands more oxygen, with glucose the preferred energy source [48]. Even so, this change might reflect an adaptative mechanism, possibly to decrease glucose uptake as seen in cases of progressive neurodegenerative diseases [49]. The metabolomic analysis of the MTX-treated brain also demonstrated a decrease in the levels of oleic acid, a monounsaturated fatty acid. Oleic acid has been reported to be released by astrocytes and behaves as a neurotrophic factor that induces neuronal differentiation [50]. Oleic acid is a ligand of the nuclear receptor TLX/NR21, which resides within neural stem cells, and controls progenitor cell self-renewal and proliferation in the hippocampal formation [51]. Considering the decrease in brain oleic acid levels caused by the MTX treatment and its importance for adult neuronal neurogenesis, it is reasonable to assume that MTX might interfere with this process. The brain levels of the metabolite 2-oleoylglycerol increase after MTX treatment. It is an endocannabinoid receptor agonist [52]. The endocannabinoid receptors belong to a complex neuromodulator system highly expressed in the CNS, which is involved in the modulation of pain sensation and inhibition of neurotransmitter release [53]. While there is still little research on the effects of 2-oleoylglycerol in the brain, an increase in the levels of this metabolite may serve to modulate the release of neurotransmitters in response to the toxic insult of MTX. Finally, MTX treatment decreased the brain levels of cholest-7-en-3-ol, (3β,5α). In the literature, there are no studies regarding this metabolite. However, brain cholesterol metabolism is essential for proper neuronal function, and dysfunction in its metabolism has been associated with neurodegenerative diseases [54]. Therefore, more research is needed to understand the effects of MTX administration on the brain’s metabolism of cholesterol.

## 4. Methods and Materials

### 4.1. Drugs and Chemicals

High-performance liquid chromatography (HPLC) grade reagents were purchased from VWR (Leuven, Belgium). MTX, methoxyamine hydrochloride (≥98%), *N*,O-bis(trimethylsilyl)trifluoroacetamide with 1% trimethylchlorosilane (BSTFA + 1% TMCS), all standards used thought out this work, and reagents used for profiling glutathione metabolism determination were purchased from Sigma-Aldrich (St. Louis, MO, USA). Isoflurane was obtained from Abbott (North Chicago, IL, USA).

### 4.2. Animals

The experiments were performed in adult male CD-1 mice aged 12 weeks obtained from Charles River Laboratories (L’Arbresle, France). The animals’ body weight ranged from 33 to 46 g. According to the literature, at 12 weeks of age the mice reach sexual maturity, corresponding to a human age of 20 years old [55,56]. Three animals were housed, at maximum, per cage (Green Line IVC Sealsafe PLUS Mouse cages) in a temperature- (22 ± 2 °C) and humidity-controlled (55 ± 10%) environment in a 12 h light–dark cycle. Mice were given a standard rodent 4RF21 certificate diet (Mucedola, Settimo Milanese, Italy). Water and food were provided *ad libitum*.

The housing and experimental treatment of the animals followed the guidelines defined by the European Council Directive (2010/63/EU) transposed into Portuguese law (Decreto-Lei no. 113/2013). The experiments were performed with the approval of the Portuguese National Authority for Animal Health (DGAV, ref. 021322 of 26 October 2016) and the local Committee Responsible for Animal Welfare (ORBEA ref. 140/2015).

### 4.3. Experimental Protocol

All animals were allowed a week of acclimatization to the environment and the handler to minimize stress and improve animal welfare. Then, the experimental protocol was designed to mimic human anticancer therapy, as MTX is usually administered in cycles of multiple administrations [57]. The mice received a total cumulative dose of 6 mg/kg of MTX, given intraperitoneally (i.p.), biweekly for three weeks. MTX was dissolved in sterile sodium chloride (NaCl) 0.9%, while the control group received injections of NaCl 0.9% at the same volume and time points as the treatment group. The total cumulative dose of MTX was equivalent to a human dose of 36.3 mg/m^2^ [19]. Moreover, the administrations were performed in the afternoon, since it has been described that mice are less susceptible to the toxicity of MTX in that period of the day [58], and we aimed to decrease suffering and death. Throughout the experiment, the mice’s well-being was monitored, as previously described by our group [19,59].

After the last administration, the animals were kept for a drug-free period of two weeks. Then, mice were anaesthetized in a chamber enriched with 5% isoflurane until full sedation. When no stimuli were noted, exsanguination was conducted. The mice were decapitated, and the brain was carefully taken after cranium removal. The brain was divided into two hemispheres by the middle line. Both hemispheres were immediately frozen at −80 °C until further use.

### 4.4. Determination of Glutathione Metabolism

The quantification was performed as previously described [24]. Briefly, the right brain hemispheres, weighing 0.180 to 0.300 g, were homogenized in 400 mL of iced phosphate-buffered saline (1x). From the obtained homogenized, 50 µL was used for the quantification of RSSP pools, another 50 µL was used for the LMWT fractions and also a total of 10 µL was used for protein quantification.

The total thiol fraction (protein bound RSSP + LMTW free reduced form (RSH) + LMWT oxidized, RSSR) was achieved by reducing the sulfhydryl groups with tris(2-carboxyethyl)phosphine hydrochloride (TCEP; 100 g/L). Following 30 min of incubation at room temperature, protein precipitation was conducted by adding to the samples a solution of trichloroacetic acid (TCA 100 g/L) and 1 mM ethylenediaminetetraacetic acid (EDTA). Afterwards, each sample was centrifuged at 13,000× *g* for 10 min at 4 °C. The obtained supernatant was then collected into a new tube containing 1.55 M of sodium hydroxide (NaOH) and 125 mM sodium tetraborate buffer (Na_2_B_4_O_7_, pH 9.5) with 4 mM EDTA, and 7-fluorobenzo-2-oxa-1,3-diazole-4-sulfonic acid ammonium ((SBD-F) 1 g/L) in Na2B4O7 buffer (125 mM with 4 mM EDTA)). The final mixture was incubated for 1 h at 60 °C and protected from light until full derivatization of the free sulfhydryl groups. After the waiting period, 10 µL of each sample was analysed using reverse-phase HPLC with fluorescence detection.

To obtain the LMWT fraction (RSSR + RSH), TCA was added to the homogenized samples to allow for protein precipitation, which was followed by centrifugation (13,000× *g*, 10 min, 4 °C). The supernatant was further transferred to a new tube containing the TCEP reagent and incubated for 30 min at room temperature. After the incubation period, the derivatization protocol described above was followed.

The quantifications were performed with HPLC with fluorescence detection on a Shimadzu LC-10AD VP (Shimadzu Scientific Instruments Inc) system using a reverse-phase C18 LiChroCART 250-4 column (LiChrospher 100 RP-18, 5 μm, VWR, Radnor, PA, USA), maintained at 29 °C with adaptations to the methodology reported by Nolin and colleagues [60]. Aminothiols were eluted with a mixture of 100 mM of sodium acetate with methanol (98:2) pH 4.5 buffer, in an isocratic mode at a flow rate of 0.6 mL/min for 22 min. Detection of aminothiols was achieved with excitation and emission wavelengths of 385 and 515 nm, respectively. The data were normalised to the total protein content.

### 4.5. Protein Quantification

The protein content of the collected sample was assayed with the commercial kit DC^TM^ Protein Assay (Bio-Rad^®^, Hercules, CA, USA) according to the manufacturer’s recommendations and using bovine serum albumin (BSA) as a standard.

### 4.6. Statistical Analysis Used with the Glutathiolomic Data

The results are presented as the mean ± standard deviation (SD), and a *t*-test analysis with Welch’s correction was used to compare the MTX-treated and the control groups. The statistical analyses were conducted using GraphPad Prism version 8.0.2 (GraphPad Software, San Diego, CA, USA), and statistical significance was accepted at *p* < 0.05 and considered a tendency whenever *p* > 0.05 but *p* < 0.1.

### 4.7. Sample Preparation for Untargeted Metabolomics Analysis

The samples for the metabolomics analysis were prepared following the protocol of Araújo and colleagues [61] with minor changes, as described below. For this analysis, the left brain hemisphere was used as stated before. For each 10 mg of brain, 150 µL of ultrapure water was added followed by 30 s of sonication, maintaining the tube samples always on ice. From the obtained homogenate, 835 µL was transferred to a new tube containing 334 µL of ice-cold methanol (MeOH). The mixture was centrifuged (16,000× *g*, 10 min, 4 °C), and the supernatant was further collected in a glass vial. A volume of 334 µL of ice-cold chloroform (CHCl_3_) was added to the pellet and placed agitated on ice for 30 min. Afterwards, the samples were centrifuged (16,000× *g*, 10 min, 4 °C), and the supernatant was added to the previous glass vial. The samples were evaporated to dryness under a gentle static stream of nitrogen at room temperature. For derivatization, 50 µL of methoxyamine hydrochloride in pyridine (15 mg/mL) was added to each sample and incubated for 1 h at 70 °C. Then, 100 µL BSTFA with 1% TMCS was added to each sample and incubated for 1 h at room temperature. The derivatized residues were transferred to an autosampler vial and analysed using gas chromatography-mass spectrometry (GC-MS).

### 4.8. Chromatographic and Mass Spectrometry Conditions

The endogenous metabolites were analysed in an EVOQ 436 GC system (Bruker Daltonics, Fremont, CA, USA) coupled to a SCION Triple Quadrupole mass detector, with a capillary column, Rxi-5Sil MS (30 m × 0.25 mm × 0.25 μm), from RESTEK. A total of 2 µL of sample was injected in the split mode (ratio 1:20), and helium C-60 (Gasin, Perafita, Portugal) was the carrier gas used at a constant flow rate (1.0 mL/min). The injector port was heated at 250 °C (maintained for 20 min). The oven temperature was fixed at 70 °C for 2 min and then gradually increased to 250 °C (rate 15 °C/min), maintained for 2 min, followed by a gradual increase to 300 °C (rate 10 °C/min) and maintained for 5 min. The total chromatographic run time was 26 min. The MS detector was operated in the electron ionization (EI) mode (70 eV). The transfer line, manifold, and EI temperature were 280 °C, 40 °C, and 270 °C, respectively. Data acquisition was performed in the full scan mode with a mass range between 50 and 500 *m*/*z* at a scan rate of 8 scans/s. The samples were analysed as a single batch to prevent analytical bias, and the preparation and data acquisition were randomized.

### 4.9. Pre-Processing and Statistical Analysis of GC-MS Data

The GC-MS files were converted to NetCDF format and uploaded to the MZmine 2.23 software [62]. The data were processed through several steps before analysis. Firstly, crop filtering was applied to restrict the mass-to-charge (*m*/*z*) range to 50–500 and retention time (RT) range to 4.35–25.11 min. Next, an asymmetric baseline corrector was used for baseline correction. Then, peak detection was performed with the noise level set at 4.0 × 10^5^. Deconvolution was also applied, with a minimum peak height of 1.4 × 10^6^, a baseline level of 3.0 × 10^5^, and a peak duration range of 0.02–0.25 min. Finally, alignment was conducted with an *m*/*z* tolerance of 0.1 and an RT tolerance of 0.05. After these pre-processing steps, the data were normalized by total chromatogram area and log-transformed to eliminate any systematic bias. Any artefact peaks from the chromatographic column, chromatographic peaks with a signal-to-noise ratio less than three, as well as any peaks with a relative standard deviation (RSD) higher than 30% across each sample group were manually removed from the data matrix. The obtained matrix was imported to the software SIMCA-P 13 (Umetrics, Umea, Sweden). The scaling method Pareto was applied since it presented the best-explained variance (R^2^) and predictive capability (Q^2^) for the partial least squares discriminant analysis (PLS-DA) model. The model is more robust for Q^2^ values closer to 1, with a value ≥ 0.4 being acceptable for biological samples [63]. PLS-DA was performed to discriminate groups and identify metabolic signatures associated with each group. The reliability of the PLS-DA model was verified using a thorough approach, which included a sevenfold internal cross-validation process. This process involved extracting R^2^ and Q^2^ values and subjecting the model to permutation tests (500 permutations). After the PLS-DA analysis, all variables that presented variable importance to the projection (VIP) > 1 and |*p*(corr)| > 0.5 were considered potentially discriminant metabolites. The statistical significance of the variations was assessed by univariate analysis (unpaired *t*-test with Welch’s correction for normal distribution or the unpaired Mann–Whitney test for non-normal distribution) (GraphPad Prism, version 8, San Diego, CA, USA). Furthermore, for each significant discriminant metabolite, the magnitude of variation in the samples of MTX-treated mice relative to controls was evaluated by calculating the effect size (ES) [64] and the percentage of variation.

### 4.10. Identification of Potentially Discriminant Metabolites

According to the Metabolomics Standards Initiative guidelines [65], the potentially discriminant metabolites were categorized into four levels of identification. The retention index (RI), RT, and mass spectrum of the sample chromatographic peaks were compared with the metabolites present in the National Institute of Standards and Technology (NIST14) mass spectral library. The identification of possible discriminant metabolites was conducted only for forward and reverse percentages of a match equal to or higher than 70%. When available, reference standards were injected under the same conditions as the samples to unmistakably confirm the identity of the metabolite.

### 4.11. Metabolic Pathway Analysis

To identify the metabolic pathways affected by MTX, all significantly altered metabolites (*p*-value < 0.05) or with a tendency (0.05 < *p*-value < 0.1) were analysed using the MetboAnalyst 5.0 (https://www.metaboanalyst.ca (accessed on 23 March 2023)) and searched against the *Mus musculus* (SMPDB) database pathways. The specific pathway analysis parameters included the hypergeometric test and out-degree centrality, and when a *p*-value was lower than 0.1, the biochemical pathways were selected for biological interpretation.

## 5. Conclusions

The goal of this study was to assess brain AOP disruption due to the administration of a clinically relevant cumulative dose of MTX in adult CD-1 mice. To the best of our knowledge, this study brings novelty on AOPs of the neurotoxic effects of MTX in vivo with the added value of using a protocol that mimics clinical cycles of administrations. Although MTX is considered not to cross an intact BBB, it significantly disrupted the glutathione metabolism, and the metabolic analysis revealed several crucial pathways altered, including phosphatidylethanolamine, ascorbic acid, and unsaturated fatty acids biosynthesis and glycerolipids metabolism. Further investigation is needed to access the impact of MTX in these pathways, particularly to access structural neuronal integrity and the levels of neurotransmitters, neuroplasticity, and neurogenesis, and to relate them with our data. However, this study is a steppingstone to understanding the cognitive deficit effects caused by chemotherapeutic treatments and the neurotoxic pathways involved, as well as toward providing better care for chemotherapy-treated patients.

## Figures and Tables

**Table 1:** List of metabolites found significantly altered in MTX-treated mice compared to control mice.

Metabolite	Effect Size ± SE	Variation ± Uncertainty (%)	*p*-Value	Down- or Upregulated	HMDB Identification	Potential Biochemical Pathway *	ID Level **
L-5-oxoproline	17.81 ± 6.6	1.30 ± 0.58	0.0519	↑	HMDB0000267	-	L2
Phosphorylethanolamine	49.86 ± 10.5	1.79 ± 0.63	*p* < 0.05	↑	HMDB0000224	Phosphatidylethanolamine Biosynthesis	L2
Ascorbic acid	36.52 ± 10.1	1.49 ± 0.59	*p* < 0.05	↑	HMDB0000044	Catecholamine Biosynthesis	L2
Palmitic acid	−23.11 ± 8.7	−1.61 ± 0.61	*p* < 0.05	↓	HMDB0000220	Glycerolipid Metabolism	L1
Linoleic acid	−24.71 ± 12.0	−1.27 ± 0.57	*p* < 0.05	↓	HMDB0000673	Biosynthesis of unsaturated fatty acids	L1
Oleic acid	−22.92 ± 11.5	−1.21 ± 0.57	0.0798	↓	HMDB0000207	-	L1
2-Oleoylglycerol	39.93 ± 14.1	1.15 ± 0.56	0.0584	↑	HMDB0011537	-	L2
Cholest-7-en-3-ol, (3β,5α)	−19.26 ± 7.7	−1.45 ± 0.59	*p* < 0.05	↓	-	-	L2

* Pathway analysis suggested by the software MetaboAnalyst 5.0. Only biochemical pathways with a *p*-value < 0.1 were considered. ** ID level categorization is provided in the Supplementary Materials. ↓ means downregulated ↑ means upregulated.

## Data Availability

The data presented in this study are available upon request from the corresponding authors.

## Supplementary Materials

The supporting information can be downloaded at: https://www.mdpi.com/article/10.3390/ijms241713126/s1.

## Abbreviations

BBB	Blood–brain barrier
CNS	Central Nervous System
Cys	Cysteine
CysGly	Cysteinylglycine
ES	Effect size
GC-MS	Gas chromatography-mass spectrometry
GSH	Glutathione
HPLC	High-performance liquid chromatography
LMWT	Low-molecular weight thiols
MTX	Mitoxantrone
PLS-DA	Partial least squares discriminant analysis
ROS	Reactive oxygen species
RSSP	*S*-thiolated proteins
RT	Retention time
SD	Standard deviation
