# Supplementary material for: A Clinically Relevant Dosage of Mitoxantrone Disrupts the Glutathione and Lipid Metabolic Pathways of the CD-1 Mice Brain: A Metabolomics Study
Source: Int J Mol Sci. 2023 Aug 23;24(17):13126. doi: 10.3390/ijms241713126 (PMC10488007; doi:10.3390/ijms241713126)
Supplement: Supplementary file 1 [file ijms-24-13126-s001.zip › ijms-2480585-supplementary.pdf]

Supplementary data

Table S1: Potential discriminant metabolites identified in the obtained matrix according to the PLS-DA scattering plots (VIP> 1 and |p(corr)|>0.05. The identification of the metabolites was performed following the Metabolomics Standards Initiative guidelines. They were characterized by retention time (RT), characteristic ions (m/z), retention index [from the literature (RI_{lit}) and compared with the calculated (RI_{calc}) for the same chromatographic column] and HMDB code when available. Only for forward and reverse percentages of match equal or higher than 700, the putative identification was considered.

**Metabolite**	**RT (min)**	**Characteristic ions (*m/z*)**	RI_{lit}	RI_{calc}	***HMDB code***	***ID level***
Phosphoric acid monomethyl ester	4.865	241+73+133+242+256	1170	1153	HMDB0061835	L2
unknown 1	5.642	192+191+73+117+163	--	--	--	L4
Erythrono-1,4-lactone	7.401	147+73+101+247+75	1383	1365	HMDB0000349	L2
L-5-oxoproline	8.897	156+73+147+157+258	1522	1510	HMDB0000267	L2
D-ribose	10.193	73+103+147+217+307	1668	1663	HMDB0000283	L2
Phosphorylethanolamine	10.987	73+299+188+174+172	NA	1768	HMDB0000224	L2
Citric acid	11.242	273+73+147+363+375	1845	1803	HMDB0000094	L1
unknown 2	11.466	73+173+157+147+75	--	--	--	L4
Ascorbic acid	12.137	73+147+332+205+117	1971	1932	HMDB0000044	L2
Scyllo-inositol	12.661	73+147+318+217+305	2043	2012	HMDB0006088	L2
Palmitic acid	12.808	313+73+117+75+129	2050	2035	HMDB0000220	L1
Myo-inositol	13.07	73+147+217+305+191	2074	2076	HMDB0000211	L1
9-Octadecenoic acid (Z)-, methyl ester	13.178	55+74+69+67+83	2091	2093	--	L2
Methyl stearate	13.332	74+87+55+143+57	2128	2119	HMDB0034154	L2
Linoleic acid	13.834	73+75+67+55+81	2212	2202	HMDB0000673	L1
Oleic acid	13.864	73+75+117+55+129	2218	2207	HMDB0000207	L1
Stearic acid	14.027	117+73+341+75+132	2246	2233	HMDB0000827	L1
Arachidonic acid methyl ester	14.142	79+67+91+80+55	2274	2251	--	L2
Oleamide	14.859	59+72+55+69+67	2386	2354	HMDB0002117	L1
5-methyluridine	15.507	73+217+103+259+147	2478	2432	HMDB0000884	L2
Docosahexaenoic Acid	16.556	73+79+91+117+75	2562	2545	HMDB0002183	L2

Inosine	16.672	73+217+230+245+281	2562	2558	HMDB0000195	L2
2-Oleoylglycerol	18.007	73+103+129+55+67	2744	2707	HMDB0011537	L2
glycerol monostearate	18.501	399+73+147+400+57	2767	2767	HMDB0011535	L2
Cholest-7-en-3-ol, (3β,5α)	21.704	75+255+458+107+73	3195	3190	--	L2
Deoxycholic acid	22.306	255+73+75+147+129	3212	3257	HMDB0000626	L2

Notes: L1 (Level 1) – Metabolites unequivocally identified by standards; L2 (Level 2) – Putatively annotated compounds (i.e., identification was based only in the mass spectrum similarity with commercial spectral libraries, retention indexes and reverse percentage of match);L4 (Level 4) – Unknown compounds (although unidentified or unclassified metabolites can still be differentiated based upon mass spectrum data). According to proposed standards by Sumner et al. 2007.
